# Highly enhanced gas sensing in single-walled carbon nanotube-based thin-film transistor sensors by ultraviolet light irradiation

**DOI:** 10.1186/1556-276X-7-644

**Published:** 2012-11-23

**Authors:** Tingting Chen, Liangming Wei, Zhihua Zhou, Diwen Shi, Jian Wang, Jiang Zhao, Yuan Yu, Ying Wang, Yafei Zhang

**Affiliations:** 1 Key Laboratory for Thin Film and Microfabrication of the Ministry of Education, Institute of Micro/Nano Science and Technology, Shanghai Jiao Tong University, Shanghai, 200240, People's Republic of China; 2 State Key Laboratory of Electronic Thin Film and Integrated Devices, School of Microelectronics and Solid-State Electronics, University of Electronic Science and Technology of China, Chengdu, 610054, China

**Keywords:** Single-walled carbon nanotubes, Gas sensor, UV radiation, Thin-film transistor

## Abstract

Single-walled carbon nanotube (SWCNT) random networks are easily fabricated on a wafer scale, which provides an attractive path to large-scale SWCNT-based thin-film transistor (TFT) manufacturing. However, the mixture of semiconducting SWCNTs and metallic SWCNTs (m-SWCNTs) in the networks significantly limits the TFT performance due to the m-SWCNTs dominating the charge transport. In this paper, we have achieved a uniform and high-density SWCNT network throughout a complete 3-in. Si/SiO_2_ wafer using a solution-based assembly method. We further utilized UV radiation to etch m-SWCNTs from the networks, and a remarkable increase in the channel current on/off ratio (*I*_on_/*I*_off_) from 11 to 5.6 × 10^3^ was observed. Furthermore, we used the SWCNT-TFTs as gas sensors to detect methyl methylphosphonate, a stimulant of benchmark threats. It was found that the SWCNT-TFT sensors treated with UV radiation show a much higher sensitivity and faster response to the analytes than those without treatment with UV radiation.

## Background

Recently, there are many reports on the use of single-walled carbon nanotubes (SWCNTs) for fabricating a chemical sensor [1]. The SWCNTs with every atom on the surface are expected to exhibit excellent sensitivity toward absorbates [2,3]. SWCNTs also possess good environmental stability, excellent electronic properties, and ultrahigh ratio of surface to volume [4]. These features make SWCNTs ideal sensing materials for compact, low-cost, low-power, and potable chemical sensors [5]. Multiple types of SWCNT-based chemical sensors, such as chemiresistors [6,7], chemicapacitors [8], and field-effect transistors (FETs) [9], have been developed for sensing application, but the SWCNT-FETs offer several advantages for sensing including the ability to amplify the detection signal with the additional gate electrode [10]. Since Kong et al. first made use of SWCNT-FETs to detect NO_2_ and NH_3_[11], the SWCNT-FETs have been successfully used to detect a variety of gases or chemical vapors, such as formaldehyde [12], O_2_[13], organophosphor [6,7], and TNT vapors [14]. It has been shown that the SWCNT-FETs exhibit significant conductance change upon exposure to absorbed molecules. Despite these significant progresses made in this field, the fabrication of high-performance SWCNT thin-film transistor (TFT) sensors still faces a big challenge due to the coexistence of metallic and semiconducting nanotubes (denoted as m-SWCNTs and s-SWCNTs, respectively) within the conducting channels [10]. In these unsorted SWCNT-TFTs, the electrical properties of the SWCNT networks are dominated by the m-SWCNTs in which small shifts of the Fermi level do not result in a substantial change in the density of state at the Fermi level, thus providing a lower electronic response upon analyte interaction with nanotubes [10,11].

Several approaches have been developed for obtaining SWCNT-FETs with pure s-SWCNT in the channels such as selective synthesis of s-SWCNTs [15-18], post-treatment [19-21], and selective etching of m-SWCNTs [22,23]. Gomez et al. have reported the method of using light irradiation to induce the metal-to-semiconductor conversion of SWCNTs for transistors based on both aligned and individual nanotube devices [24]. This method increased the channel current on/off ratio up to 10^5^ in the SWCNT transistors. Roberts et al. reported a self-sorting method to enrich and align the s-SWCNT on the surface of silicon and polymeric films by controlling the substrate surface chemistry [25]. It was found that the aligned nanotube networks enriched with s-SWCNT showed a much higher sensitivity to analytes than those fabricated with random networks.

Random nanotube networks are easily deposited from the solution and represent an attractive path to large-scale device manufacturing [26-28]. However, they suffer from the mixture of s-SWCNTs and m-SWCNTs in the network, often resulting in a low on/off ratio due to the m-SWCNTs dominating the charge transport. Wang et al. [27] reported on the deposition of uniform and high-density s-SWCNT random networks from a s-SWCNT solution onto a wafer using an assembly method. These s-SWCNT-TFTs show on/off ratios larger than 10^4^. However, this technology requires pre-separating 95% enriched s-SWCNTs. Here, we reported on the deposition of random SWCNT networks from the SWCNT solution on the wafer and then radiated the nanotube networks utilizing UV to fabricate SWCNT-TFT sensors with high performance. A remarkable increase in the channel current on/off ratio from approximately 11 to 5.6 × 10^3^ was observed after UV irradiation. We also used these SWCNT-TFTs to detect methyl methylphosphonate (DMMP), a stimulant of benchmark threats. The SWCNT-TFTs treated with UV radiation show a much higher sensitivity to the analytes than those without treatment with UV radiation.

## Methods

### Materials

The carboxylic acid-functionalized SWCNTs were obtained from Carbon Solutions Inc. (Riverside, CA, USA). The Si/SiO_2_ wafer used is an n-doped Si (100) wafer with 300-nm oxide layer on the top of the Si wafer. DMMP and 3-aminopropyltrimethysilane (APS) were purchased from Sigma-Aldrich (St. Louis, MO, USA). Deionized water, ethanol, acetone, and toluene were used throughout all processes for cleaning. All reagents were analytical grade and were used without further purification.

### SWCNT network deposition and sensor fabrication

To fabricate our gas sensors, the Si/SiO_2_ wafer substrate was ultrasonically rinsed with toluene, acetone, ethanol, and deionized water, followed by cleaning with a piranha solution (98% H_2_SO_4_:30% H_2_O_2_ = 3:1 (*v*/*v*)) for 2 h at 80°C. This clean wafer was immersed in APS aqueous solution (1.5 mM) for 2 h and then kept in a vacuum evaporator at 120°C for 1 h to form the amino-terminated monolayer on the surface of the Si/SiO_2_ substrate. The purified SWCNTs were ultrasonically dispersed in deionized water for 2 h. The pretreated Si/SiO_2_ substrate modified with an APS monolayer was immersed in the SWCNT suspension, followed by rinsing with ethanol and deionized water and drying with the aid of nitrogen flow. The morphology of the deposited SWCNTs was characterized by scanning electron microscopy (SEM; JSM-7401F, JEOL Ltd., Akishima, Tokyo, Japan).

The SWCNT-TFT sensors were fabricated using the standard microfabrication procedures in our previous report [7]. The interdigitated electrode fingers were made by sputtering 10 nm Cr and 180 nm Au onto the patterned photoresist mold. We introduced a lift-off process to remove the photoresist. Finally, the electrodes were ultrasonically rinsed in acetone and ethanol repeatedly, washed with deionized water thoroughly, and then dried by nitrogen flow before they were used. The UV light radiation wavelength utilized is 250 to approximately 360 nm, and the SWCNT-TFT sensor was exposed to the UV light for 0 to 1 h with a distance of 10 cm.

### Sensor testing system

The homemade sensor testing system was established as in the previous reports [7]. Nitrogen, used as carrier gas, flowed through a porous glass-disc bubbler containing liquid DMMP to form DMMP vapor, and then, this DMMP vapor was mixed with the diluting N_2_ in a stainless steel. The output flow ratio of the diluted DMMP vapor was controlled by mass flow controllers. The different concentrations of DMMP were produced by regulating the flow ratio of the dilution gas to the flow rate of the carrier gas. The DMMP vapor was delivered into the sensing chip to test the sensor performance. Before testing, the sensor was cleaned by UV for about 2 min to remove the absorbed impurity from the SWCNT network (meanwhile, the conductance was monitored to avoid etching SWCNTs) and then exposed to the DMMP.

The electrical signal of the sensor was monitored using a semiconductor parameter analyzer (Agilent 4156C, Agilent Technologies Inc., Santa Clara, CA, USA). After a stable baseline electrical signal was obtained, the DMMP vapor with the required concentration was introduced, and all sensing measurements were carried out at room temperature. The DMMP was desorbed from the SWNT surface by N_2_ blowing together with illumination with a lamp (the wavelength was 710 nm, and the power was 200 W).

## Results and discussion

**Figure 1:**
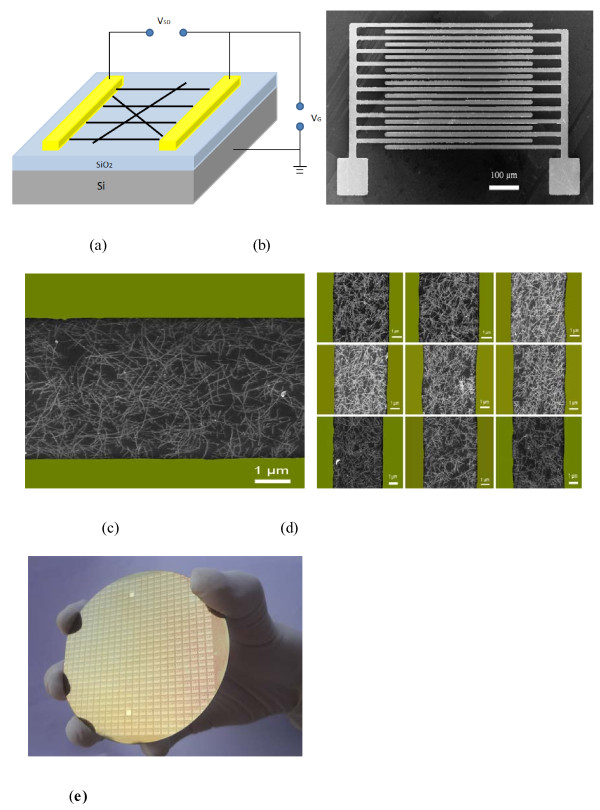
**Schematic and SEM images.** The (**a**) schematic and (**b**) SEM image of the SWCNT-TFT structure. (**c**) SEM image of SWCNT bridging the conducting channel. (**d**) SEM images showing SWCNTs deposited at different positions on the (**e**) electron patterning wafer.

**Figure 2:**
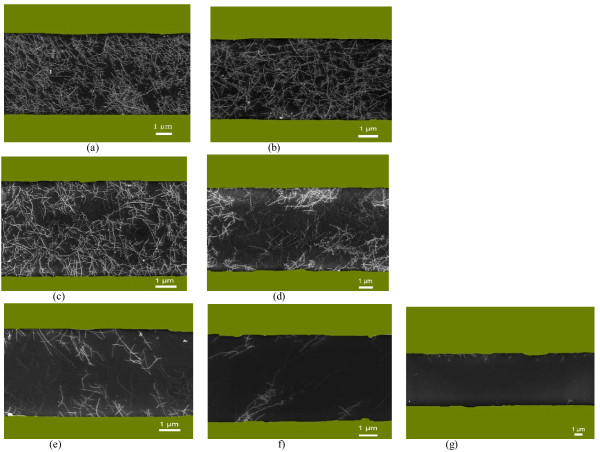
**SEM image of SWCNT networks before and after UV irradiation.** (**a**) Before UV irradiation; after (**b**) 5, (**c**) 10, (**d**) 20, (**e**) 30, (**f**) 40, and (**g**) 60 min of UV irradiation.

The uniform and high-density SWCNT networks on the Si/SiO_2_ wafer were fabricated with a solution-based self-assembly method [7,27]. Briefly, the Si/SiO_2_ substrate was first functionalized with an amino-terminated monolayer. The carboxylic acid-functionalized SWCNTs de-bounded and dispersed in the water were easily absorbed and deposited on the amino-functionalized Si/SiO_2_ substrate via electrostatic interaction. Following nanotube deposition was the fabrication of SWCNT-TFTs. The source and drain electrodes were patterned by standard photolithography. The silicon substrate acts as a back gate, while SiO_2_ is used as the back gate dielectric. The schematic and SEM images of the SWCNT-TFT structure were shown in Figure 1a,b respectively. The electrodes possess a channel length of 600 μm and a channel width of 10 μm. The SEM images show that the high density and uniform deposition of SWCNT networks bridge the source and the drain electrodes (Figure 1c). We also took the SEM images at nine different locations on the wafer to determine the deposition uniformity of the SWCNT networks (Figure 1d,e). It can be seen that the uniform and high-density nanotube networks were achieved throughout the 3-in. wafer, suggesting that the solution deposition approach is feasible to manufacture devices on a large scale. Figure 2 shows the SEM images of the SWCNT networks after irradiation by UV in air. It was found that the nanotube density decreased with an increase of the irradiation time, and at last, the nanotubes were completely removed from the wafer when a 60-min irradiation was applied.

**Figure 3:**
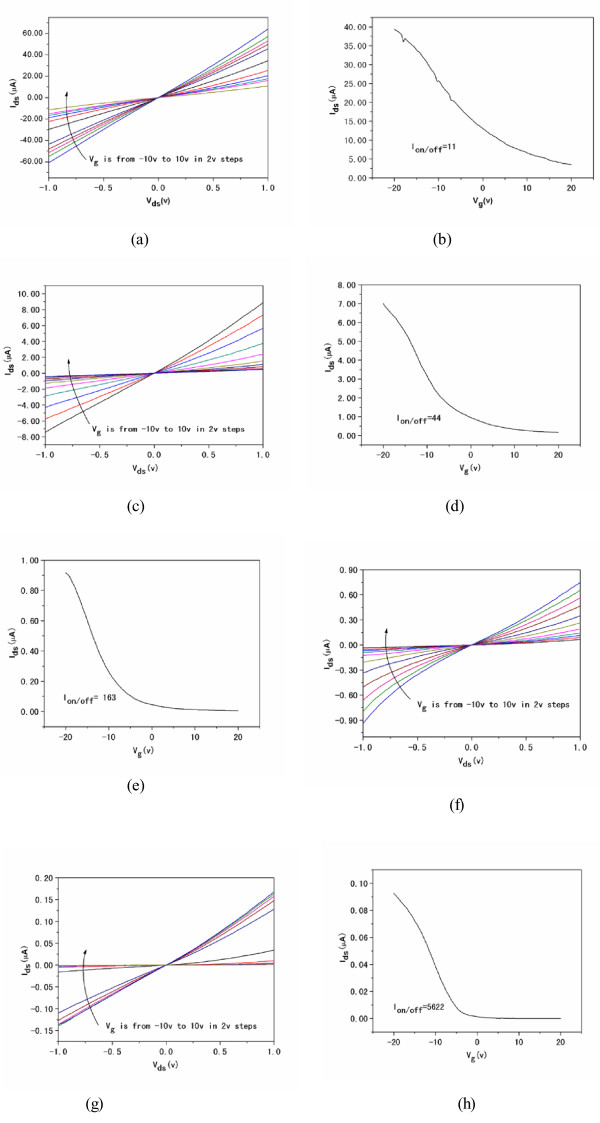
***I***_**ds **_**versus *V***_**ds **_**plots at different gate voltages (*V***_**g**_**) for SWCNT-TFT gas sensors. ***I*_ds_ versus *V*_ds_ plots after (**a**) 0, (**c**) 5, (**e**) 10, and (**g**) 20 min UV radiation (*V*_g_ is varied from −10 to 10 V in 2-V steps). The corresponding *I*_ds_-*V*_g_ plots of the SWCNT-TFTs after (**b**) 0, (**d**) 5, (**f**) 10, and (**h**) 20 min of UV radiation (*V*_ds_ = 0.5 V, *V*_g_ = −20 to approximately 20 V).

We study the electrical performance of the SWCNT-TFTs. Figure 3 shows the source-drain current current (*I*_ds_)-source-drain voltage (*V*_ds_) curves of typical SWCNT-TFTs before and after UV radiation. The *I*_ds_*V*_ds_ curves were found to be very linear for *V*_ds_ between −1 and 1 V, indicating the Ohmic contact between nanotubes and the electrodes (Figure 3a) [14,27]. After irradiation for 5 to 20 min, the *I*_ds_*V*_ds_ curves remain linear, but a significant decrease in the current was observed because nanotubes were etched from the channels by UV radiation. The SWCNT-TFTs completely lost the conductance after 30 min of illumination.

The transfer characteristic (*I*_ds_ versus *V*_g_) of the sensor was measured using the Si substrate as a back gate. The measurement was operated at a constant source-drain voltage of 0.5 V and gate voltage between −20 and +20 V. As shown in Figure 3, the SWCNT device shows a p-type semiconductor behavior with holes as the majority carriers, evidenced by the decreasing electrical conductance when gate voltage (*V*_g_) was swept from negative to positive values. The on/off ratio of the untreated SWCNT-TFTs is low (approximately 11) due to the contribution of the m-SWCNTs. After 5 to 20 min of light irradiation, the SWCNT-TFTs retained a p-type semiconductor behavior, but the on/off ratios increased from approximately 11 to approximately 5.6 × 10^3^, suggesting that most of the m-SWCNTs were etched from the channels.

**Figure 4:**
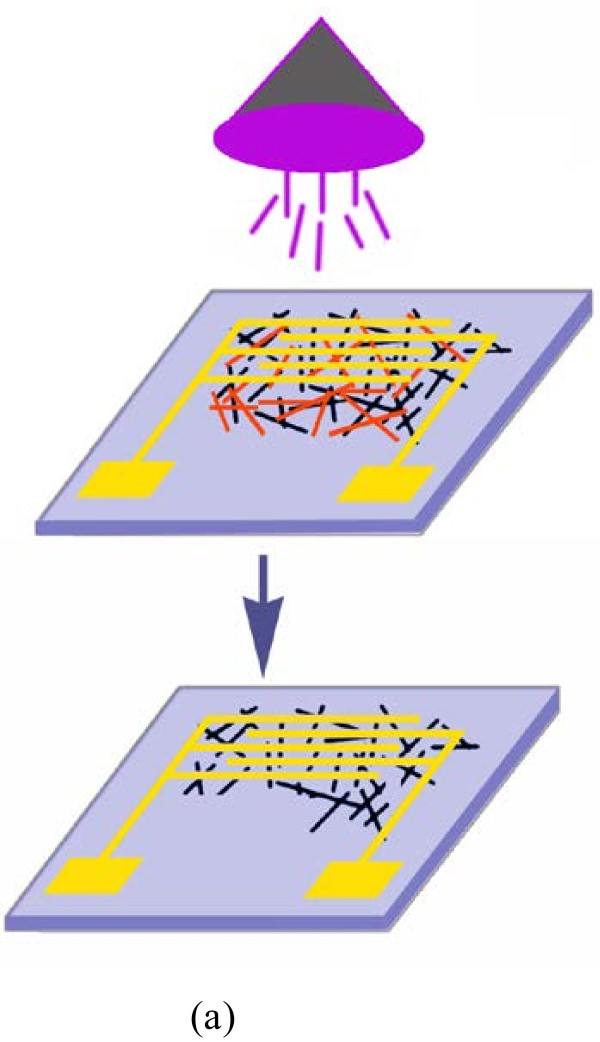
The schematic diagram for etching of m-SWCNTs from SWCNT networks.

The energy absorbed by SWCNTs is related to the light wavelength and intensity. The theoretical calculation has confirmed that the formation energy of m-SWCNTs is higher than that of the same-diameter s-SWCNTs [29,30] and that the m-SWCNTs show a higher chemical reactivity due to more abundant delocalized electronic states [31]. UV irradiation in air generated oxygen radicals and ozone, which are strong gas-phase oxidants [32]. The m-SWCNTs were easily oxidized by these oxidants and removed from the wafers as shown in Figure 4[31,32]. The UV-assisted photo-oxidation could also increase defect density on the sidewall of nanotubes due to the increase in the *sp*^3^ nature of irradiated nanotubes [24]. It is known that the conversion of *sp*^2^ to *sp*^3^ sites may lead to π-electron localization that can open the bandgap of metallic nanotubes and result in the conversion of m-SWCNTs to s-SWCNTs [24]. Both of the possible reasons might account for the high on/off ratio of the UV-irradiated SWCNT-TFT devices. However, the significant decrease in the nanotube density as shown in the SEM images suggested that the etching of m-SWCNTs from the networks may be the main reason for the high performance of TFT devices.

**Figure 5:**
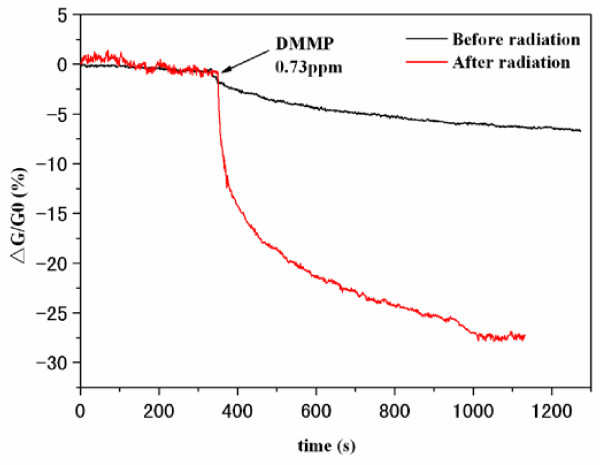
**Conductance change versus time recorded with the sensor responses to 0.73 ppm of DMMP.** Without (black) and with 20 min of UV radiation (red). *I*_ds_ = 0.5 V, *V*_g_ = 0 V.

**Figure 6:**
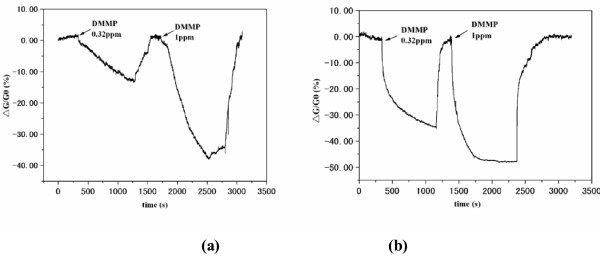
**Conductance change versus time recorded with the sensor responses to 0.32 ppm of DMMP.** (**a**) Without and (**b**) with 20 min of UV radiation. *I*_ds_ = 1 V, *V*_g_ = −10 V.

The SWCNT-TFTs were used to detect DMMP, a stimulant of benchmark threats. The conductance between two electrodes was measured to investigate the sensory response, and the change in conductance (*R*) is defined as *R* = Δ*G*/*G*_0_ = (*G* − G_0_)/G_0_, where *G*_0_ and *G* are the conductance of nanotubes before and after exposure to the testing gas, respectively. Figure 5 shows the responses of the SWCNT networks to 0.73 ppm of DMMP at a constant source-drain voltage of 0.5 V and gate voltage of 0 V. It can be seen that the resistance of the device increased upon exposure to 0.73 ppm of DMMP, and the conductance change was 17% (with a response time of 2 min) for the irradiated sensors, compared to only 2.2% conductance change for non-irradiated sensors. The irradiated SWCNT sensors show seven times higher sensitivity than those without irradiation. The sensor responses were also investigated when the SWCNT-TFTs were operated at gate voltage of −10 V at the same concentration of DMMP (Figure 6). A pronounced increase in the sensitivity of SWCNT-TFTs was also observed after the SWCNT network was irradiated with UV light. For instance, the irradiated SWCNT-TFT sensor gave a 22% conductance change upon exposure to 0.32 ppm of DMMP, whereas only 2% conductance change was observed for the unirradiated sensor under the same concentration of DMMP. The reason for these different response behaviors is that for the unirradiated SWCNTs, the electrical properties of the SWCNT network are dominated by the m-SWCNTs in which small shifts of the Fermi level do not result in a substantial change in the density of state at the Fermi level and, thus, in the charge carried in the nanotubes [10,11]. After radiation, the channel conductance was dominated by s-SWCNT, from which a donation or injection of one electron could significantly change the conductance of SWCNTs, leading to a fast response and high sensitivity toward analytes. DMMP is a typical electron-donating molecule, and s-SWCNTs are p-type semiconductors with holes as the majority carriers in the nanotubes. Adsorption of DMMP onto the s-SWCNT leads to a charge transfer from the DMMP molecule to the nanotubes [9]. This charge transfer reduced the holes carried in the nanotubes and decreased sample conductance. Figure 6 also shows that applying a negative gate bias (*V*_g_ = −10V) can enhance the sensitivity. As an electron donator, the adsorption of DMMP on the p-type SWCNTs is equivalent to applying a positive chemical gate voltage. From the *I*_ds_*V*_g_ curve (Figure 3h), a sharp slope was observed at this gate bias (−10 V). So, a large response is expected due to the positive chemical gate effect of the DMMP adsorption.

## Conclusions

We reported the deposition of uniform and high-density SWCNT networks from the SWCNT solution on the wafer and then used these nanotube networks to fabricate SWCNT-TFT sensors. UV irradiation was utilized to etch m-SWCNTs and (or) convert m-SWCNTs to s-SWCNTs. A remarkable increase in the channel current on/off ratio from approximately 11 to 5.6 × 10^3^ was observed after UV irradiation. We also used these SWCNT-TFT sensors to detect DMMP. It was found that the irradiated SWCNT-TFT sensors show a much higher sensitivity to the analytes than those without treatment with UV radiation. Our work provides a simple and efficient approach to a large-scale fabrication of SWCNT-TFT sensors and solves the problem of low on/off ratio in the nanotube-based TFTs when the random SWCNT network is used.

## Competing interests

The authors declare that they have no competing interests.

## Authors’ contributions

TTC performed the experiment and measured the SEM, electronic, and gas response data. LMW designed the experiments and wrote the manuscript. DWS helped in the technical support for the experiments. YY participated in the measurements. JW and JZ provided useful suggestions. ZHZ and YW helped analyze the characterization results. YFZ supervised all of the study and provided financial support. All authors read and approved the final manuscript.
